# Prevalence and determinants of obesity and abdominal obesity among rural workers in Southeastern Brazil

**DOI:** 10.1371/journal.pone.0270233

**Published:** 2022-07-07

**Authors:** Monica Cattafesta, Glenda Blaser Petarli, Eliana Zandonade, Olívia Maria de Paula Alves Bezerra, Sandra Marlene Ribeiro de Abreu, Luciane Bresciani Salaroli

**Affiliations:** 1 Graduate Program in Collective Health, Federal University of Espírito Santo, Vitória/ES, Brazil; 2 Department of Family Medicine, Mental and Collective Health, Federal University of Ouro Preto, Ouro Preto/MG, Brazil; 3 Research Center in Physical Activity, Health and Leisure (CIAFEL) of Faculty of Sports-University of Porto (FADEUP), Laboratory for Integrative and Translational Research in Population Health (ITR), and Faculty of Sports, Research Center on Physical Activity, Health and Leisure, University of Porto, Porto, Portugal; PLOS ONE, UNITED KINGDOM

## Abstract

The objectives of this study were to assess the nutritional status of rural workers from a municipality in Southeastern Brazil and estimate the association of sociodemographic, labor, lifestyle, and dietary pattern factors with obesity and abdominal obesity of men and women of this rural area. This is a cross-sectional, epidemiological study of 740 farmers (51.5%, n = 381 males; 48.5%, n = 359 females). The sociodemographic, labor, lifestyle and dietary patterns determinants were assessed. Food intake data were obtained by applying three 24-hour recalls and dietary patterns were determined by Principal Component Analysis with Varimax orthogonal rotation. Poisson regression with robust variance stratified by sex was applied. The general prevalence of overweight status was 31.5% (95% CI 28.2–34.8%), 19.7% of obesity (95% CI 16.8–22.6%) and 31.5% of abdominal obesity (95% CI 28.2–34.8%), with higher rates in women (*P* < 0.001). Men of higher socioeconomic class had a 2.3 times higher prevalence of obesity (95% CI 1.08–4.90). In addition, the shorter travel time to purchase food increased the prevalence of abdominal obesity in males. For women, the older the age group, the greater the general and central obesity. A lower adherence to traditional dietary patterns (approximately PR [prevalence ratio] 1.6 for general obesity and PR 1.3 for abdominal obesity) and a greater number of places to buy food were associated with higher rates of obesity in women. Finally, women farmers with a higher workload had a 20% lower prevalence of central obesity (PR 0.80; 95% CI 0.65–0.97). Such findings demonstrate that obesity must be an issue in the health care of remote and rural populations. There is a need to promote healthier environments that respect traditional food culture through multiple approaches that consider the heterogeneity of rural areas and the differences between sexes.

## Introduction

Obesity is considered a disease, multicausal and chronic, characterized by excessive accumulation of fat [1], an uncontrolled pandemic across the globe [1, 2]. Increasingly rising, this morbidity is a challenge for the health infrastructure and service delivery systems of countries, especially developing countries [3], given its relationship with chronic noncommunicable diseases (NCDs) [4, 5].

According to the World Health Organization (WHO), there is convincing evidence that both general obesity, assessed by the body mass index (BMI), and abdominal obesity, assessed by the waist circumference (WC), are related to a higher risk of cardiovascular disease, type 2 diabetes mellitus, high blood pressure, general mortality and some types of cancer [6]. Furthermore, these anthropometric measures can be complementary, since abdominal obesity presents itself as a more sensitive marker for metabolic diseases [7–9]. In addition to increasing the risk of all-cause mortality for stroke, diabetes and cardiovascular diseases [6, 8–11], obesity has other implications for health, quality of life and productivity, burdening the public sectors [12]. Globally, the current costs of obesity are estimated at around US $2 trillion per year, both in direct costs of health services and for the loss of economic productivity, which represents 2.8% of the world gross domestic product (GDP) [5].

In Brazil, the situation is no different, as it is among the three countries with the highest growth in obesity cases in the world [13], with successive increases of BMI in recent years [14]. The change in the nutritional profile is evidenced in Brazilian surveys. In the 45 years between 1974–1975 to 2019, the prevalence of overweightness in adult Brazilian population practically tripled in males (from 18.5% to 60.0%) and doubled in females (from 28.7% to 63.3%). In addition, obesity has vertiginously increased both in men (from 2.8% to 22.8%) and women (from 8.0% to 30.2%) [15, 16]. Data from the National Health Survey also demonstrate a continuous growth of overweight status, with a general average prevalence of 25.9% for obesity in 2019 [16]. The prevalence of abdominal obesity is also even greater, affecting 37.7% of the Brazilian population, with rates much higher in women (52.1%) than men (21.8%) [17].

It is a fact that body weight has been considerably increasing in most countries, mainly in developed countries and urban areas [18]. However, data from the NCD Risk Factor Collaboration 2019 reported a more than 55% global increase in average BMI from 1985 to 2017, and more than 80% in some low- and middle-income regions, which occurred due to the increase in BMI in rural areas, challenging the view that the rise in obesity has been an exclusively urban problem. With the exception of women in sub-Saharan Africa, this rate is increasing at the same or faster level in rural areas as in cities in low- and middle-income regions, characterizing weight gain in rural areas as the main driver of the obesity epidemic in the contemporary world [19].

Despite this, the multicausal character of this endocrine, nutritional and metabolic disease makes it difficult to control, since several factors are involved in its genesis, such as environmental factors, represented by the political, economic, physical, social and perceived spheres in which the individual would be inserted, as well as biological, metabolic and individual factors [20]. Furthermore, it is observed that, in both urban and rural areas, urbanization is the important driving force of obesity [12, 19, 21] due to changes in the global food system [22]. However, rural areas differ from urban areas in many aspects that are difficult to measure in population studies, such as sociodemographic, socioeconomic and cultural factors [23], and therefore, surveys that evaluate these particularities are necessary. Additionally, labor aspects in these rural areas have been minimally explored, even though it is a possible determinant of obesity in rural areas [24–26].

The objectives of this study were to assess the nutritional status of rural workers from a municipality in Southeastern Brazil and to estimate the association of sociodemographic, labor, lifestyle, and dietary pattern factors with obesity and abdominal obesity of men and women in this rural area.

## Materials and methods

### Study design

This is an epidemiological study of cross-sectional, analytical and quantitative design developed in the municipality of Santa Maria de Jetibá, Espírito Santo, Southeastern Brazil. This study integrates, in a larger scope, the study “Health condition and associated factors: a study in farmers of Espírito Santo–AgroSaúdES” [27–33].

The representative sample of male and female farmers met the following inclusion criteria: adults 20–59 years old, non-pregnant, with agriculture as their main source of income and in full employment for at least six months. Individuals who did not meet these criteria were excluded. To identify eligible farmers, we used data available in the records of individuals and families conducted by the Family Health Strategy teams, responsible for covering 100% of the eleven health regions of the municipality.

### Sampling

The population universe of this study included 7,287 farmers distributed among 4,018 families. In this population, we calculated a minimum sample of 708 farmers, considering a sampling error of 3.5%, 95% confidence interval and an estimated prevalence of 50.2% overweightness in the rural Brazilian population [17]. In order to reach the minimum sample and considering possible losses, recruitment included 806 individuals. Of the 806 individuals invited to participate, 790 underwent data collection. Of these, 50 individuals were excluded since they did not complete the data collection on food consumption (6.3% loss), resulting in a final sample of 740 farmers. As a result, the total was above the minimum sample of 708 farmers; therefore, the group was considered representative of the total population.

To define the sample universe, one list was built with a survey of the registration of individuals and families by the community health agents, through the data available in the family register used by the Family Health Strategy teams. The participants were selected by stratified draw proportional to the number of families per health region in order to respect proportionality among the eleven health regions. In families with more than one eligible member, only one individual was drawn; thus, avoiding the interdependence of information. In cases of refusal of participation or non-attendance during data collection, a new participant on the waiting list of the lottery was called, respecting the sex and region of origin of the dropout.

### Data collection

Data collection took place between December 2016 and April 2017 in the facilities of the municipal health units of the Family Health Strategy teams. A semi-structured questionnaire with questions about socioeconomic, labor, lifestyle characteristics, food consumption, and anthropometric was utilized [34].

### Independent variables

The independent variables of this study were subdivided into sociodemographic, labor, lifestyle, and dietary patterns.

Among the sociodemographic variables evaluated were sex, age group (“up to 29 years”, “30 to 39 years”, “40 to 49 years” and “50 years or more”), marital status (“single”, “married/living with a partner” and “divorced/separated/widowed”), race/color (“white” and “non-white”), schooling (“less than 4 years”, “4 to 8 years” and “more than 8 years”), land bond (“owner” and “non-owner”), transport used more frequently (“own vehicle” and “on foot, by bicycle or bus”), nearby places for physical activity (“there is no proper place” and “around the house”), and socioeconomic class (“A or B”, “C” and “D or E”). These designations were assigned according to the Criteria of Economic Classification Brazil, which has used in national studies to estimate socioeconomic classes according to the purchasing power of individuals and families, projecting the average monthly gross family income (“E” is the lowest socioeconomic class, to “A”, the highest socioeconomic class) [35].

Labor variables were investigated by questioning working time as a farmer (“under 10 years”, “from 10 to 29 years” and “30 years or more”), the current type of production (“conventional” and “non-conventional”), the number of worked crops (“up to 4 crops” and “5 or more crops”), the type of worked crops categorized into “temporary only”, “permanent only” and “temporary and permanent” (according to the criteria of the Brazilian Institute of Geography and Statistics [36]), the workload (hours/week) (“less than or equal to 40 hours” and “more than 40 hours”) and contact with pesticides (“direct contact” and “indirect contact, organic or agroecological”) [37].

Lifestyle variables included alcohol consumption, categorized as “non-drinking”, for individuals who reported not consuming alcoholic beverages, and “drinking”, for individuals who reported consuming alcoholic beverages; smoking, assessed according to the Smoker Approach and Treatment Consensus and categorized as “non-smoker” and “current and past smoker”; practice of physical activity extra-field (“yes” or “no”); and screen time, obtained by the sum of daily activities for television, video game and computer/cell phone, divided by the days of the week, classified as “no sedentary leisure” when < two hours/day and “with sedentary leisure” when ≥ two hours/day [38]. Also evaluated were the number of places where participants usually buy food (“2 places or less” and “3 places or more”), the frequency of food purchases (“twice/month or more” and “once/month or less”), travel time to purchase food (“up to 15 minutes”, “16 to 29 minutes” and “more than 30 minutes”), monthly per capita expenditure on food purchases (“R$ 100 or less”, “> R$ 100 to < R$ 200” and “R$ 200 or more”), the habit of eating away from home (“no or rarely” and “yes, often”) and the place where they usually eat meals (“at a table” and “under a different setting”).

Dietary patterns were determined using the principal component analysis (PCA) method, as published by Cattafesta et al. [30]. Food consumption was obtained by applying three 24-hour recalls (R24h) (two days of the week and one day of the weekend).

The nutritional composition of the R24h was performed using the software AvaNutri 4.1 (Avanutri Equipamentos de Avaliação Ltda, Três Rios, Rio de Janeiro, Brazil), in which the Brazilian Table of Food Composition [39] was selected for extraction of nutritional information. After obtaining the values of each R24h, the analysis of the attenuation was performed using the PC-SIDE software (Department of Statistics, Iowa State University, Iowa, United States of America), which follows the methodology of Nusser et al. [40]. After registering the food and acquiring the caloric counts, no exclusion was performed due to extremes in energy consumption [41].

In total, 355 different food items reported in the R24h were listed. From these items, 65 foods were removed for not constituting the eating habits of the analyzed population [42, 43]. The remaining foods were allocated to 25 groups according to their nutritional characteristics and Pearson’s correlation between their food items [42, 44] and the applicability of the PCA method was evaluated by the Kaiser-Meyer-Olkin coefficient (KMO) and Bartlett’s test of sphericity (BTS) [45]. According to Cattel graph analysis, three patterns were then extracted, selecting Varimax rotation to obtain uncorrelated factors [45]. In this way, three dietary patterns were obtained: “local traditional” (with sugar, coffee, butter and margarine, homemade bread, cakes and cookies, juice and sugary beverages, potatoes, yams and cassava, and pasta), “traditional Brazilian” (with beans, rice, vegetables, flour, and oils and fats), and “industrialized” (with soda, snacks, fried foods, hamburgers, hot dogs, garlic bread and trooper’s beans, red meats, sausage, canned food, industrialized condiments and sauces, alcoholic drinks, and industrialized breads, cookies, toasts and threads). Adherence to dietary patterns was evaluated in quartiles. The “least adhesion” to the “local traditional” and “traditional Brazilian” dietary patterns were considered for the first quartile of adherence to these patterns. Nonetheless, the “largest adhesion” to the “industrialized” dietary pattern was considered for the fourth quartile in this pattern [30].

### Dependent variables

Obesity was assessed according to the BMI, and the presence of abdominal obesity was measured by the WC. Weight was measured using the Omron514C® digital scale (Omron Healthcare Brasil, São Paulo, São Paulo, Brazil), with a capacity of 150 kg and precision of 0.1 kg. Height was measured with a Sanny portable ES-2060® stadiometer (Promohealth Trade in Medical and Specialized Products, Bauru, São Paulo, Brazil) to the nearest 0.1 mm. Participants were positioned on the scale with their arms extended along their bodies, with as few clothes as possible, after emptying their bladder [46]. For the collection of WC, an inextensible Sanny tape measure TR-4010® (Promohealth Trade in Medical and Specialized Products, Bauru, São Paulo, Brazil) was used. The subject was instructed to remain standing, with the arms extended along the body and the feet together. The tape was positioned at the smallest curvature, located between the last costal arch and the iliac crest [46]. When it was impossible to visualize the smallest curvature, the midpoint between these two anatomical points was used as a reference [47]. For all measures, there were three repetitions, non-consecutive, with the first discarded and the average of the last two considered the final measure.

From these data, BMI was calculated (BMI = weight [kg]/height [m]^2^) and classified according to the WHO [1] into categories of underweight (BMI < 18.5 kg/m^2^), eutrophic (18.5–24.9 kg/m^2^), overweight (25–29.9 kg/m^2^) and obesity (≥ 30 kg/m^2^). After this general assessment of nutritional status, to determine the prevalence of obesity and its determinants, this classification was recategorized into “no obesity” (BMI < 30.0 kg/m^2^) and “obesity” (BMI ≥ 30 kg/m^2^). WC was also classified according to the WHO [1] and categorized as “no abdominal obesity” for WC ≤ 102 cm for men and ≤ 88 cm for women, and “abdominal obesity” for values above these.

### Statistical analysis

Absolute and percentage values were used to describe the study variables. Regarding the association tests between the independent variables and the outcomes for the qualitative variables, Pearson’s Chi-square test was used. When the expected values in the table cells were less than five or when the sum of the column value was less than twenty, Fisher’s exact test was used.

The Poisson regression with robust variance stratified by sex was applied to assess the association between independent variables and obesity and abdominal obesity. Variables that were statistically significant with obesity indices of up to 20% in the association analyses were tested in multiple models. In addition, the calorie consumption was used as an adjustment variable for these final models. The absence of multicollinearity (tolerance > 0.1 and variance inflation factor < 10), minimum sample size for the number of model variables, and absence of outliers were also evaluated.

We based ourselves on the relevance of the applicability of the prevalence ratio (PR) for the following reasons: 1) because it is an article whose objective was to work with prevalences and, therefore, the Poisson regression presents the data because of prevalence, we opted for this analysis; 2) by the prevalence found to be 19.7% of obesity and 31.5% of abdominal obesity. In cross-sectional studies with very prevalent outcomes (intermediate to high prevalence, that is, greater than 10%), the use of Poisson Regression is recommended, since the odds ratio (OR) overestimates the risk [48]; 3) the comparability of our results with other large national studies that used RP instead of OR [26, 49–51].

For all analyses, the level of significance adopted was α < 5% and these were performed using the statistical software IBM SPSS Statistics for Windows, version 22.0 (IBM Corp, Armonk, New York, United States of America).

### Ethical standards disclosure

This study was conducted according to the guidelines laid down in the Declaration of Helsinki and all procedures involving research study participants were approved by the Research Ethics Committee of the Health Sciences Center of the Federal University of Espírito Santo (Ufes) under number 1,856,331 (CAAE 52839116.3.0000.5060). Written informed consent was obtained from all subjects/patients.

## Results

### General characteristics of study population

Of the 740 farmers evaluated, 51.5% (n = 381) was men and 48.5% (n = 359) was women. Most of the evaluated farmers were married or living with a partner (86.2%, n = 638), were in socioeconomic class C (50.8%, n = 376), self classifies as white race/color (90.4%, n = 669), and had low level schooling (67.7% with less than four years of schooling) (Table 1). When assessing the difference by sex, the proportion of single men was higher in relation to women (*P* < 0.001), as well as the socioeconomic class A or B (*P* < 0.001).

**Table 1:** General characteristics of study population, by sex.

Variables	Sex		*P*-value	Total
	Male	Female		n (%)
	n (%)	n (%)		
**Age group**			0.956	
Up to 29 years	101 (26.5)	100 (27.9)		201 (27.2)
30 to 39 years	114 (29.9)	104 (28.9)		218 (29.5)
40 to 49 years	93 (24.4)	90 (25.1)		183 (24.7)
50 years or more	73 (19.2)	65 (18.1)		138 (18.6)
**Marital status**			**<0.001**	
Single	46 (12.1)	10 (2.8)		56 (7.6)
Married/living with a partner	321 (84.3)	317 (88.3)		638 (86.2)
Divorced/separated/widowed	14 (3.7)	32 (8.9)		46 (6.2)
**Race/color**			0.174	
White	339 (89.0)	330 (91.9)		669 (90.4)
Non-white	42 (11.0)	29 (8.1)		71 (9.6)
**Socioeconomic class**			**<0.001**	
A or B	41 (10.8)	15 (4.2)		56 (7.6)
C	208 (54.6)	168 (46.8)		376 (50.8)
D or E	132 (34.6)	176 (49.0)		308 (41.6)
**Schooling**			0.655	
Less than 4 years	253 (66.4)	248 (69.1)		501 (67.7)
4 to 8 years	88 (23.1)	73 (20.3)		161 (21.8)
More than 8 years	40 (10.5)	38 (10.6)		78 (10.5)

n, individuals number; socioeconomic class according to the Criteria of Economic Classification Brazil used in national studies to estimate socioeconomic classes according to the purchasing power of individuals and families, projecting the average monthly gross family income, therefore, “E” (lowest socioeconomic class) to “A” (highest socioeconomic class).

Chi-square test. n = 740.

### Prevalence of obesity and abdominal obesity

The general prevalence of overweight status was 31.5% (95% CI = 28.2–34.8%) and obesity was 19.7% (95% CI = 16.8–22.6%), reaching 51.2% of excess weight (95% CI = 47.6–54.8%) (Table 2). Abdominal obesity was observed in 31.5% (95% CI = 28.2–34.8%) of individuals. It is noteworthy that underweight status was present in only 1.5% (95% CI = 0.6–2.4%, n = 11) of the sample (males, n = 3; females, n = 8).

**Table 2:** Nutritional status and prevalence of general and abdominal obesity in a rural Brazilian area, according to sex, age group, marital status, race/color, socioeconomic class and schooling.

Variables	BMI (n = 740)										WC (n = 739)						
	Non-overweight			Overweight			Obesity			*P*-value	No abdominal obesity			Abdominal obesity			*P*-value
	n	%	95% CI	n	%	95% CI	n	%	95% CI		n	%	95% CI	n	%	95% CI	
**All subjects**	361	48.8	(45.2–52.4)	233	**31.5**	**(28.2–34.8)**	**146**	**19.7**	**(16.8–22.6)**	**<0.001**	506	68.5	(65.2–71.8)	**233**	**31.5**	**(28.2–34.8)**	**<0.001**
**Sex**										**<0.001**							**<0.001**
Male	201	52.8	(49.2–56.4)	**127**	**33.3**	**(29.9–36.7)**	**53**	**13.9**	**(11.4–16.4)**		326	85.8	(83.3–88.3)	**54**	**14.2**	**(11.7–16.7)**	
Female	160	44.6	(41.0–48.2)	**106**	**29.5**	**(26.2–32.8)**	**93**	**25.9**	**(22.7–29.1)**		180	50.1	(46.5–53.7)	**179**	**49.9**	**(46.3–53.5)**	
**Age group**										**<0.001**							**<0.001**
Up to 29 years	130	64.7	(61.3–68.1)	50	24.9	(21.8–28.0)	21	10.4	(8.2–12.6)		168	83.6	(80.9–86.3)	33	16.4	(13.7–19.1)	
30 to 39 years	107	49.1	(45.5–52.7)	70	32.1	(28.7–35.5)	41	18.8	(16.0–21.6)		153	70.2	(66.9–73.5)	65	29.8	(26.5–33.1)	
40 to 49 years	80	43.7	(40.1–47.3)	57	31.1	(27.8–34.4)	46	25.1	(22.0–28.2)		110	60.4	(56.9–63.9)	72	39.6	(36.1–43.1)	
50 years or more	44	31.9	(28.5–35.3)	**56**	**40.6**	**(37.1–44.1)**	**38**	**27.5**	**(24.3–30.7)**		75	54.3	(50.7–57.9)	**63**	**45.7**	**(42.1–49.3)**	
**Marital status**										0.350							**0.002**
Single	34	60.7	(57.2–64.2)	14	25.0	(21.9–28.1)	8	14.3	(11.8–16.8)		46	82.1	(79.3–84.9)	10	17.9	(15.1–20.7)	
Married/living with a partner	304	47.6	(44.0–51.2)	207	32.4	(29.0–35.8)	127	19.9	(17.0–22.8)		437	68.6	(65.3–71.9)	200	31.4	(28.1–34.7)	
Divorced/separated/widowed	23	50.0	(46.2–53.6)	12	26.1	(22.9–29.3)	11	23.9	(20.8–27.0)		23	50.0	(46.4–53.6)	**23**	**50.0**	**(46.4–53.6)**	
**Race/color**										0.549							0.522
White	322	48.1	(44.5–51.7)	213	31.8	(28.4–35.2)	134	20.0	(17.1–22.9)		455	68.1	(64.7–71.5)	213	31.9	(28.5–35.3)	
Non-white	39	54.9	(51.3–58.5)	20	28.2	(25.0–31.4)	12	16.9	(14.2–19.6)		51	71.8	(68.6–75.0)	20	28.2	(25.0–31.4)	
**Socioeconomic class**										0.319							0.814
A or B	26	46.4	(42.8–50.0)	15	26.8	(23.6–30.0)	15	26.8	(23.6–30.0)		40	71.4	(68.1–74.7)	16	28.6	(25.3–31.9)	
C	192	51.1	(47.5–54.7)	110	29.3	(26.0–32.6)	74	19.7	(16.8–22.6)		259	68.9	(65.6–72.2)	117	31.1	(27.8–34.4)	
D or E	143	46.4	(42.8–50.0)	108	35.1	(31.7–38.5)	57	18.5	(15.7–21.3)		207	67.4	(64.0–70.8)	100	32.6	(29.2–36.0)	
**Schooling**										**<0.001**							**0.002**
Less than 4 years	215	42.9	(39.3–46.5)	**174**	**34.7**	**(31.3–38.1)**	**112**	**22.4**	**(19.4–25.4)**		322	64.4	(61.0–67.8)	**178**	**35.6**	**(32.2–39.0)**	
4 to 8 years	94	58.4	(54.8–62.0)	44	27.3	(24.1–30.5)	23	14.3	(11.8–16.8)		126	78.3	(75.3–81.3)	35	21.7	(18.7–24.7)	
More than 8 years	52	66.7	(63.3–70.1)	15	19.2	(16.4–22.0)	11	14.1	(11.6–16.6)		58	74.4	(71.3–77.5)	20	25.6	(22.5–28.7)	

BMI, Body mass index; WC, Waist circumference; n, individuals number; 95% CI, 95% confidence interval.

Chi-square test.

When the prevalence of obesity and abdominal obesity by sex was assessed, a prevalence of 33.3% of overweight (95% CI = 29.9–36.7%) and 13.9% (95% CI = 11.4–16.4%) of obesity males were found. In women, the prevalence of overweight status was 29.5% (95% CI = 26.2–32.8%) and obesity was 25.9% (95% CI = 22.7–29.1%); therefore, with higher rates in women (*P* < 0.001). Evaluating the sum of these figures, overweight statuses (overweight and obesity) affected 47.2% (95% CI = 43.6–50.8%) of men and 55.4% (95% CI = 51.8–59.0%) of women. When assessing abdominal obesity, rates were much higher in females (*P* < 0.001), with 49.9% (95% CI = 46.3–53.5%) presenting abdominal obesity, in contrast to 14.2% (95% CI = 11.7–16.7%) of men.

A higher prevalence of overweight status (*P* < 0.001), obesity (*P* < 0.001) and abdominal obesity (*P* < 0.001) were identified in individuals aged 50 years or older. Rural workers who were divorced, separated or widowed also had a higher prevalence of abdominal obesity (*P* = 0.002). Workers with less than four years of study also showed higher overweight status (*P* < 0.001), obesity (*P* < 0.001) and abdominal obesity (*P* = 0.002). There were no differences in the prevalence of general and abdominal obesity according to the study participant race/color and socioeconomic class.

### Factors associated with obesity and abdominal obesity, stratified by sex

When assessing the factors associated with obesity, stratified by sex, it was possible to identify in men that obesity was higher among individuals in socioeconomic class A or B (*P* = 0.023), as well as in those who worked 30 years or more as a farmer (*P* = 0.024) and who worked with non-conventional production (*P* = 0.024). In women, a greater number of factors proved to be associated with obesity, among them age over 40 years (*P* < 0.001), low schooling level (*P* = 0.020), the most frequent transport not being an own vehicle (*P* = 0.003), working for 30 years or more as a farmer (*P* = 0.001), being a current or recurrent smoker (*P* = 0.027) and adhering less to the traditional local dietary pattern (*P* = 0.003) (Table 3).

**Table 3:** General obesity and sociodemographic, labor, lifestyle and dietary pattern variables of a rural Brazilian population, according to sex.

Variable	Male (n = 381)			Female (n = 359)		
	No obesity	Obesity	*P*-value	No obesity	Obesity	*P*-value
	n (%)	n (%)		n (%)	n (%)	
**Age group**			0.170			**<0.001**
Up to 29 years	91 (90.1)	10 (9.9)		89 (89.0)	11 (11.0)	
30 to 39 years	101 (88.6)	13 (11.4)		76 (73.1)	28 (26.9)	
40 to 49 years	78 (83.9)	15 (16.1)		**59 (65.6)**	**31 (34.4)**	
50 years or more	58 (79.5)	15 (20.5)		**42 (64.6)**	**23 (35.4)**	
**Marital status**			0.401*			0.563*
Single	42 (91.3)	4 (8.7)		6 (60.0)	4 (40.0)	
Married/living with a partner	275 (85.7)	46 (14.3)		236 (74.4)	81 (25.6)	
Divorced/separated/widowed	11 (78.6)	3 (21.4)		24 (75.0)	8 (25.0)	
**Race/color**			0.690			0.821
White	291 (85.8)	48 (14.2)		244 (73.9)	86 (26.1)	
Non-white	37 (88.1)	5 (11.9)		22 (75.9)	7 (24.1)	
**Socioeconomic class**			**0.023**			0.912*
A or B	**30 (73.2)**	**11 (26.8)**		11 (73.3)	4 (26.7)	
C	179 (86.1)	29 (13.9)		123 (73.2)	45 (26.8)	
D or E	119 (90.2)	13 (9.8)		132 (75.0)	44 (25.0)	
**Schooling**			0.852			**0.020**
Less than 4 years	216 (85.4)	37 (14.6)		**173 (69.8)**	**75 (30.2)**	
4 to 8 years	77 (87.5)	11 (12.5)		61 (83.6)	12 (16.4)	
More than 8 years	35 (87.5)	5 (12.5)		32 (84.2)	6 (15.8)	
**Land bond**			0.560			0.880
Owner	265 (86.6)	41 (13.4)		201 (73.9)	71 (26.1)	
Non-owner	63 (84.0)	12 (16.0)		65 (74.7)	22 (25.3)	
**Transport used more frequently** ^a^			0.999*			**0.003**
Own vehicle	317 (85.9)	52 (14.1)		**245 (76.3)**	**76 (23.7)**	
On foot, by bicycle or bus	10 (90.9)	1 (9.1)		20 (54.1)	17 (45.9)	
**Nearby places for physical activity**			0.169			0.711
There is no proper place	190 (84.1)	36 (15.9)		195 (73.6)	70 (26.4)	
Around the house	138 (89.0)	17 (11.0)		71 (75.5)	23 (24.5)	
**Working time as a farmer** ^a^			0.020*			0.001*
Under 10 years	20 (100.0)	0 (0.0)		12 (80.0)	3 (20.0)	
From 10 to 29 years	172 (88.7)	22 (11.3)		148 (81.8)	33 (18.2)	
30 years or more	**135 (81.3)**	**31 (18.7)**		**105 (64.8)**	**57 (35.2)**	
**Current type of production**			**0.024**			0.737
Conventional	295 (87.5)	42 (12.5)		243 (73.9)	86 (26.1)	
Non-conventional	**33 (75.0)**	**11 (25.0)**		23 (76.7)	7 (23.3)	
**Number of worked crops**			0.508			0.873
Up to 4 crops	127 (87.6)	18 (12.4)		129 (73.7)	46 (26.3)	
5 or more crops	201 (85.2)	35 (14.8)		137 (74.5)	47 (25.5)	
**Type of worked crops**			0.612*			0.489
Temporary only	154 (87.5)	22 (12.5)		138 (76.2)	43 (23.8)	
Permanent only	20 (90.9)	2 (9.1)		18 (78.3)	5 (21.7)	
Temporary and permanent	154 (84.2)	29 (15.8)		110 (71.0)	45 (29.0)	
**Workload (hours/week)**			0.239			0.164
Less than or equal to 40 hours	32 (80.0)	8 (20.0)		74 (69.2)	33 (30.8)	
More than 40 hours	296 (86.8)	45 (13.2)		192 (76.2)	60 (23.8)	
**Contact with pesticides**			0.057			0.775
Direct contact	286 (87.5)	41 (12.5)		139 (74.7)	47 (25.3)	
Indirect contact, organic or agroecological	42 (77.8)	12 (22.2)		127 (73.4)	46 (26.6)	
**Alcohol consumption**			0.063			0.357
Non-drinking	123 (82.0)	27 (18.0)		196 (72.9)	73 (27.1)	
Drinking	205 (88.7)	26 (11.3)		70 (77.8)	20 (22.2)	
**Smoking**			0.254			0.027*
Non-smoker	247 (87.3)	36 (12.7)		260 (75.1)	86 (24.9)	
Current and past smoker	81 (82.7)	17 (17.3)		**6 (46.2)**	**7 (53.8)**	
**Physical activity extra-field**			0.755			0.136
No	260 (85.8)	43 (14.2)		229 (75.6)	74 (24.4)	
Yes	68 (87.2)	10 (12.8)		37 (66.1)	19 (33.9)	
**Screen time** ^b^			0.441			0.726
No sedentary leisure	179 (87.3)	26 (12.7)		146 (73.4)	53 (26.6)	
With sedentary leisure	148 (84.6)	27 (15.4)		120 (75.0)	40 (25.0)	
**Number of places where they usually buy food**			0.786			0.169
2 places or less	186 (86.5)	29 (13.5)		145 (77.1)	43 (22.9)	
3 places or more	142 (85.5)	24 (14.5)		121 (70.8)	50 (29.2)	
**Frequency of food purchases**			0.963			0.764
Twice/month or more	98 (86.0)	16 (14.0)		70 (75.3)	23 (24.7)	
Once/month or less	230 (86.1)	37 (13.9)		196 (73.7)	70 (26.3)	
**Travel time to purchase food** ^c^			0.175			0.858
Up to 15 minutes	98 (81.7)	22 (18.3)		66 (75.0)	22 (25.0)	
16 to 29 minutes	158 (89.3)	19 (10.7)		132 (72.9)	49 (27.1)	
More than 30 minutes	70 (86.4)	11 (13.6)		63 (75.9)	20 (24.1)	
**Monthly *per capita* expenditure on food purchases** ^d^			0.218			0.142
R$ 100 or less	120 (87.0)	18 (13.0)		105 (74.5)	36 (25.5)	
> R$ 100 to < R$ 200	145 (88.4)	19 (11.6)		120 (77.4)	35 (22.6)	
R$ 200 or more	51 (79.7)	13 (20.3)		31 (63.3)	18 (36.7)	
**Habit of eating away from home**			0.181			0.757
No or rarely	187 (88.2)	25 (11.8)		210 (74.5)	72 (25.5)	
Yes, often	141 (83.4)	28 (16.6)		56 (72.7)	21 (27.3)	
**Place where they usually meal**			0.513			0.205
At the table	243 (86.8)	37 (13.2)		194 (72.4)	74 (27.6)	
Under a different setting	85 (84.2)	16 (15.8)		72 (79.1)	19 (20.9)	
**Pattern 1 –Local traditional**			0.310			**0.003**
Largest adhesion (2^nd^ to 4^th^ quartiles)	278 (86.6)	43 (13.4)		185 (79.1)	49 (20.9)	
Least adhesion (1^st^ quartile)	50 (83.3)	10 (16.7)		**81 (64.8)**	**44 (35.2)**	
**Pattern 2 –Traditional Brazilian**			0.449			0.145
Largest adhesion (2^nd^ to 4^th^ quartiles)	272 (85.8)	45 (14.2)		181 (76.1)	57 (23.9)	
Least adhesion (1^st^ quartile)	56 (87.5)	8 (12.5)		85 (70.2)	36 (29.8)	
**Pattern 3 –Industrialized**			0.279			0.093
Least adhesion (1^st^ to 3^rd^ quartiles)	218 (85.2)	38 (14.8)		217 (72.6)	82 (27.4)	
Largest adhesion (4^th^ quartile)	110 (88.0)	15 (12.0)		49 (81.7)	11 (18.3)	

n, individuals number.

Chi-square test.

* Fisher’s Exact Test.

n = 740.

^a^ n = 738.

^b^ n = 739.

^c^ n = 730.

^d^ n = 711.

For men, being 50 years old or older (*P* = 0.005), working as a farmer for 30 years or more (*P* = 0.015), working with non-conventional production (*P* = 0.029), working up to 40 hours/week (*P* = 0.002), having indirect contact with pesticides (*P* = 0.025) and spending less than 15 minutes to buy food (*P* = 0.035) were factors associated with abdominal obesity. However, being 50 years old or more (*P* < 0.001), as well as having less than four years of study (*P* = 0.001), not using your own vehicle as the main means of transport (*P* = 0.024), working in the field for 30 years or more (*P* < 0.001), working up to 40 hours/week (*P* = 0.004), and adhering less to the traditional local dietary pattern (*P* = 0.003) and the industrialized dietary pattern (*P* = 0.034) were factors associated with women’s central obesity (Table 4).

**Table 4:** Abdominal obesity and sociodemographic, labor, lifestyle and dietary pattern variables of a rural Brazilian population, according to sex.

Variable	Male (n = 380)			Female (n = 359)		
	No abdominal obesity	Abdominal obesity	*P*-value	No abdominal obesity	Abdominal obesity	*P*-value
	n (%)	n (%)		n (%)	n (%)	
**Age group**			**0.005**			**<0.001**
Up to 29 years	94 (93.1)	7 (6.9)		74 (74.0)	26 (26.0)	
30 to 39 years	98 (86.0)	16 (14.0)		55 (52.9)	49 (47.1)	
40 to 49 years	80 (87.0)	12 (13.0)		30 (33.3)	60 (66.7)	
50 years or more	**54 (74.0)**	**19 (26.0)**		**21 (32.3)**	**44 (67.7)**	
**Marital status**			0.158*			0.398*
Single	42 (91.3)	4 (8.7)		4 (40.0)	6 (60.0)	
Married/living with a partner	274 (85.6)	46 (14.4)		163 (51.4)	154 (48.6)	
Divorced/separated/widowed	10 (71.4)	4 (28.6)		13 (40.6)	19 (59.4)	
**Race/color**			0.988			0.859
White	290 (85.8)	48 (14.2)		165 (50.0)	165 (50.0)	
Non-white	36 (85.7)	6 (14.3)		15 (51.7)	14 (48.3)	
**Socioeconomic class**			0.135			0.459
A or B	31 (75.6)	10 (24.4)		9 (60.0)	6 (40.0)	
C	180 (86.5)	28 (13.5)		79 (47.0)	89 (53.0)	
D or E	115 (87.8)	16 (12.2)		92 (52.3)	84 (47.7)	
**Schooling**			0.683			**0.001**
Less than 4 years	214 (84.9)	38 (15.1)		**108 (43.5)**	**140 (56.5)**	
4 to 8 years	78 (88.6)	10 (11.4)		48 (65.8)	25 (34.2)	
More than 8 years	34 (85.0)	6 (15.0)		24 (63.2)	14 (36.8)	
**Land bond**			0.620			0.878
Owner	263 (86.2)	42 (13.8)		137 (50.4)	135 (49.6)	
Non-owner	63 (84.0)	12 (16.0)		43 (49.4)	44 (50.6)	
**Transport used more frequently** ^a^			0.661*			**0.024**
Own vehicle	316 (85.9)	52 (14.1)		167 (52.0)	154 (48.0)	
On foot, by bicycle or bus	9 (81.8)	2 (18.2)		**12 (32.4)**	**25 (67.6)**	
**Nearby places for physical activity**			0.388			0.975
There is no proper place	191 (84.5)	35 (15.5)		133 (50.2)	132 (49.8)	
Around the house	135 (87.7)	19 (12.3)		47 (50.0)	47 (50.0)	
**Working time as a farmer** ^a^			0.015*			**<0.001**
Under 10 years	20 (100.0)	0 (0.0)		9 (60.0)	6 (40.0)	
From 10 to 29 years	172 (88.7)	22 (11.3)		119 (65.7)	62 (34.3)	
30 years or more	**133 (80.6)**	**32 (19.4)**		**51 (31.5)**	**111 (68.5)**	
**Current type of production**			**0.029**			0.715
Conventional	293 (87.2)	43 (12.8)		164 (49.8)	165 (50.2)	
Non-conventional	**33 (75.0)**	**11 (25.0)**		16 (53.3)	14 (46.7)	
**Number of worked crops**			0.642			0.225
Up to 4 crops	122 (84.7)	22 (15.3)		82 (46.9)	93 (53.1)	
5 or more crops	204 (86.4)	32 (13.6)		98 (53.3)	86 (46.7)	
**Type of worked crops**			0.837*			0.731
Temporary only	153 (86.9)	23 (13.1)		87 (48.1)	94 (51.9)	
Permanent only	19 (86.4)	3 (13.6)		12 (52.2)	11 (47.8)	
Temporary and permanent	154 (84.6)	28 (15.4)		81 (52.3)	74 (47.7)	
**Workload (hours/week)**			**0.002**			**0.004**
Less than or equal to 40 hours	**28 (70.0)**	**12 (30.0)**		**41 (38.3)**	**66 (61.7)**	
More than 40 hours	298 (87.6)	42 (12.4)		139 (55.2)	113 (44.8)	
**Contact with pesticides**			**0.025**			0.633
Direct contact	285 (87.4)	41 (12.6)		91 (48.9)	95 (51.1)	
Indirect contact, organic or agroecological	**41 (75.9)**	**13 (24.1)**		89 (51.4)	84 (48.6)	
**Alcohol consumption**			0.080			0.648
Non-drinking	122 (81.9)	27 (18.1)		133 (49.4)	136 (50.6)	
Drinking	204 (88.3)	27 (11.7)		47 (52.2)	43 (47.8)	
**Smoking**			0.455			0.391
Non-smoker	245 (86.6)	38 (13.4)		175 (50.6)	171 (49.4)	
Current and past smoker	81 (83.5)	16 (16.5)		5 (38.5)	8 (61.5)	
**Physical activity extra-field**			0.448			0.754
No	257 (85.1)	45 (14.9)		153 (50.5)	150 (49.5)	
Yes	69 (88.5)	9 (11.5)		27 (48.2)	29 (51.8)	
**Screen time** ^b^			0.951			0.310
No sedentary leisure	176 (85.9)	29 (14.1)		95 (47.7)	104 (52.3)	
With sedentary leisure	149 (85.6)	25 (14.4)		85 (53.1)	75 (46.9)	
**Number of places where they usually buy food**			0.449			0.065
2 places or less	187 (87.0)	28 (13.0)		103 (54.8)	85 (45.2)	
3 places or more	139 (84.2)	26 (15.8)		77 (45.0)	94 (55.0)	
**Frequency of food purchases**			0.369			0.568
Twice/month or more	95 (83.3)	19 (16.7)		49 (52.7)	44 (47.3)	
Once/month or less	231 (86.8)	35 (13.2)		131 (49.2)	135 (50.8)	
**Travel time to purchase food** ^c^			**0.035**			0.948
Up to 15 minutes	**95 (79.8)**	**24 (20.2)**		43 (48.9)	45 (51.1)	
16 to 29 minutes	160 (90.4)	17 (9.6)		92 (50.8)	89 (49.2)	
More than 30 minutes	70 (86.4)	11 (13.6)		41 (49.4)	42 (50.6)	
**Monthly *per capita* expenditure on food purchases** ^d^			0.621			0.124
R$ 100 or less	121 (87.7)	17 (12.3)		75 (53.2)	66 (46.8)	
> R$ 100 to < R$ 200	141 (86.0)	23 (14.0)		80 (51.6)	75 (48.4)	
R$ 200 or more	52 (82.5)	11 (17.5)		18 (36.7)	31 (63.3)	
**Habit of eating away from home**			0.378			0.354
No or rarely	184 (87.2)	27 (12.8)		145 (51.4)	137 (48.6)	
Yes, often	142 (84.0)	27 (16.0)		35 (45.5)	42 (54.5)	
**Place where they usually meal**			0.584			0.928
At the table	241 (86.4)	38 (13.6)		134 (50.0)	134 (50.0)	
Under a different setting	85 (84.2)	16 (15.8)		46 (50.5)	45 (49.5)	
**Pattern 1 –Local traditional**			0.210			**0.003**
Largest adhesion (2^nd^ to 4^th^ quartiles)	277 (86.6)	43 (13.4)		130 (55.6)	104 (44.4)	
Least adhesion (1^st^ quartile)	49 (81.7)	11 (18.3)		**50 (40.0)**	**75 (60.0)**	
**Pattern 2 –Traditional Brazilian**			0.441			0.055
Largest adhesion (2^nd^ to 4^th^ quartiles)	271 (85.5)	46 (14.5)		127 (53.4)	111 (46.6)	
Least adhesion (1^st^ quartile)	55 (87.3)	8 (12.7)		53 (43.8)	68 (56.2)	
**Pattern 3 –Industrialized**			0.242			**0.034**
Least adhesion (1^st^ to 3^rd^ quartiles)	216 (84.7)	39 (15.3)		**143 (47.8)**	**156 (52.2)**	
Largest adhesion (4^th^ quartile)	110 (88.0)	15 (12.0)		37 (61.7)	23 (38.3)	

n, individuals number.

Chi-square test.

* Fisher’s Exact Test.

n = 739.

^a^ n = 737.

^b^ n = 738.

^c^ n = 729.

^d^ n = 710.

After multiple analyses, the obesity of men remained associated only with socioeconomic class, in which individuals of class A or B had 2.3 times higher prevalence of general obesity than those of the lower classes (95% CI = 1.08–4.90) (Table 5). Moreover, the time spent to buy food (16–29 minutes) remained associated with a lower prevalence of central obesity in men (PR = 0.54, 95% CI = 0.31–0.95) (Table 6).

**Table 5:** Factors associated with general obesity among men in a rural Brazilian region.

Variables	Crude			Adjusted*		
	*P*-value	PR	95% CI	*P*-value	PR	95% CI
**Age group**						
Up to 29 years		1			1	
30 to 39 years	0.878	1.07	(0.46–2.47)	0.699	1.17	(0.53–2.59)
40 to 49 years	0.229	1.63	(0.73–3.64)	0.319	1.45	(0.70–3.02)
50 years or more	0.197	1.74	(0.75–4.02)	0.323	1.46	(0.69–3.12)
**Socioeconomic class**						
D or E		1			1	
C	0.323	1.41	(0.71–2.78)	0.420	1.30	(0.69–2.46)
A or B	**0.027**	**2.58**	**(1.12–5.98)**	**0.030**	**2.30**	**(1.08–4.90)**
**Nearby places for physical activity**						
There is no proper place		1			1	
Around the house	0.188	0.67	(0.37–1.22)	0.179	0.69	(0.40–1.19)
**Current type of production**						
Conventional		1			1	
Non-conventional	0.125	1.76	(0.85–3.63)	0.187	1.50	(0.82–2.76)
**Alcohol consumption**						
Non-drinking		1			1	
Drinking	**0.046**	**0.56**	**(0.32–0.99)**	0.061	0.61	(0.37–1.02)
Travel time to purchase food**						
Up to 15 minutes		1			1	
16 to 29 minutes	0.110	0.59	(0.31–1.12)	0.067	0.59	(0.33–1.04)
More than 30 minutes	0.545	0.80	(0.38–1.66)	0.694	0.88	(0.45–1.70)
**Habit of eating away from home**						
No or rarely		1			1	
Yes, often	0.476	1.23	(0.70–2.15)	0.336	1.28	(0.78–2.10)

PR, prevalence ratio; 95% IC, 95% confidence interval.

Poisson regression.

* Adjusted for all variables with *P* < 0.02 in the association test (age group, socioeconomic class, nearby places for physical activity, current type of production, alcohol consumption, travel time to purchase food, and habit of eating away from home) and caloric consumption. The variables “working time as a farmer” and “contact with pesticides” were not included in the model due to multicollinearity with “age group” and “current type of production”, respectively.

n = 381.

** n = 378.

**Table 6:** Factors associated with abdominal obesity among men in a rural Brazilian region.

Variables	Crude			Adjusted*		
	*P*-value	PR	95% CI	*P*-value	PR	95% CI
**Age group**						
Up to 29 years		1			1	
30 to 39 years	0.213	1.78	(0.72–4.41)	0.199	1.92	(0.71–5.19)
40 to 49 years	0.181	1.89	(0.74–4.80)	0.279	1.76	(0.63–4.86)
50 years or more	**0.008**	**3.31**	**(1.36–8.04)**	0.078	2.40	(0.91–6.38)
**Marital status**						
Single		1			1	
Married/living with a partner	0.310	0.55	(0.17–1.76)	0.431	0.66	(0.23–1.86)
Divorced/separated/ widowed	0.218	0.39	(0.09–1.74)	0.611	0.66	(0.13–3.33)
**Socioeconomic class**						
D or E		1			1	
C	0.666	1.15	(0.60–2.21)	0.659	1.15	(0.61–2.17)
A or B	0.111	1.98	(0.86–4.57)	0.108	1.91	(0.87–4.22)
**Current type of production**						
Conventional		1			1	
Non-conventional	0.127	1.76	(0.85–3.62)	0.189	1.53	(0.81–2.88)
**Workload (hours/week)**						
Less than or equal to 40 hours		1			1	
More than 40 hours	**0.014**	**0.42**	**(0.21–0.84)**	0.054	0.56	(0.31–1.01)
**Alcohol consumption**						
Non-drinking		1			1	
Drinking	**0.043**	**0.56**	**(0.32–0.98)**	0.062	0.62	(0.37–1.02)
Travel time to purchase food**						
Up to 15 minutes		1			1	
16 to 29 minutes	**0.024**	**0.48**	**(0.25–0.91)**	**0.034**	**0.54**	**(0.31–0.95)**
More than 30 minutes	0.370	0.72	(0.35–1.48)	0.847	0.94	(0.48–1.82)

PR, prevalence ratio; 95% CI, 95% confidence interval.

Poisson regression.

* Adjusted for all variables with *P* < 0.02 in the association test (age group, marital status, socioeconomic class, current type of production, workload (hours/week), alcohol consumption, and travel time to purchase food) and caloric consumption. The variables “working time as a farmer” and “contact with pesticides” were not included in the model due to multicollinearity with “age group” and “current type of production”, respectively.

n = 380.

** n = 377.

For women, the older the age group, the greater were the rates of general obesity, with up to 2.3 times the highest prevalence (95% CI = 1.04–5.08) in women aged 50 years or older. In addition, women who adhered less to a local traditional dietary pattern (PR = 1.62, 95% CI = 1.10–2.37) and the traditional Brazilian dietary pattern (PR = 1.57, 95% CI = 1.06–2.32) had a higher prevalence of obesity (Table 7). Likewise, older women with greater abdominal obesity, and those aged 50 years or older, had approximately twice the prevalence in comparison to younger women (PR = 2.16, 95% CI = 1.41–3.33). However, female farmers who worked more than 40 hours/week had a 20% lower prevalence of obesity (PR = 0.80, 95% CI = 0.65–0.97). Furthermore, women who bought food in three places or more had 1.24 times higher prevalence of abdominal obesity (95% CI = 1.01–1.51) and those who adhered less to local and Brazilian dietary patterns, had approximately 1.3 times higher prevalence of abdominal obesity (PR = 1.27, 95% CI = 1.02–1.57 for the local traditional dietary pattern and PR = 1.34, 95% CI = 1.07–1.67 for the traditional Brazilian dietary pattern) (Table 8).

**Table 7:** Factors associated with general obesity among women in a rural Brazilian region.

Variables	Crude			Adjusted*		
	*P*-value	PR	95% CI	*P*-value	PR	95% CI
**Age group**						
Up to 29 years		1			1	
30 to 39 years	**0.014**	**2.40**	**(1.19–4.85)**	**0.024**	**2.18**	**(1.11–4.27)**
40 to 49 years	**0.003**	**2.94**	**(1.46–5.93)**	**0.035**	**2.20**	**(1.06–4.60)**
50 years or more	**0.001**	**3.23**	**(1.57–6.67)**	**0.039**	**2.30**	**(1.04–5.08)**
**Schooling**						
Less than 4 years		1			1	
4 to 8 years	0.061	0.54	(0.29–1.03)	0.463	0.79	(0.43–1.47)
More than 8 years	0.128	0.52	(0.23–1.20)	0.546	0.78	(0.36–1.72)
Transport used more frequently**						
Own vehicle		1			1	
On foot, by bicycle or bus	0.029	1.86	(1.06–3.24)	0.075	1.52	(0.96–2.42)
**Workload (hours/week)**						
Less than or equal to 40 hours		1			1	
More than 40 hours	0.359	0.81	(0.52–1.27)	0.635	0.91	(0.63–1.32)
**Smoking**						
Non-smoker		1			1	
Current and past smoker	0.100	2.01	(0.88–4.60)	0.257	1.44	(0.77–2.69)
**Physical activity extra-field**						
No		1			1	
Yes	0.290	1.33	(0.78–2.26)	0.381	1.22	(0.78–1.89)
**Number of places where they usually buy food**						
2 places or less		1			1	
3 places or more	0.292	1.25	(0.82–1.91)	0.154	1.29	(0.91–1.83)
**Monthly *per capita* expenditure on food purchases** ***						
R$ 100 or less		1			1	
> R$ 100 to < R$ 200	0.542	0.86	(0.54–1.38)	0.273	0.80	(0.54–1.19)
R$ 200 or more	0.194	1.46	(0.83–2.57)	0.745	1.09	(0.66–1.78)
**Pattern 1 –Local traditional**						
Largest adhesion (2^nd^ to 4^th^ quartiles)		1			1	
Least adhesion (1^st^ quartile)	**0.004**	**1.67**	**(1.17–2.37)**	**0.014**	**1.62**	**(1.10–2.37)**
**Pattern 2 –Traditional Brazilian**						
Largest adhesion (2^nd^ to 4^th^ quartiles)		1			1	
Least adhesion (1^st^ quartile)	0.062	1.41	(0.98–2.01)	**0.024**	**1.57**	**(1.06–2.32)**
**Pattern 3 –Industrialized**						
Least adhesion (1^st^ to 3^rd^ quartiles)		1			1	
Largest adhesion (4^th^ quartile)	0.263	0.73	(0.41–1.27)	0.574	0.83	(0.44–1.57)

PR, prevalence ratio; 95% CI, 95% confidence interval.

Poisson regression.

* Adjusted for all variables with *P* < 0.02 in the association test (age group, schooling, transport used more frequently, workload (hours/week), smoking, physical activity extra-field, number of places where they usually buy food, monthly *per capita* expenditure on food purchases, dietary pattern 1 –Local traditional, dietary pattern 2 –Traditional Brazilian, and dietary pattern 3 –Industrialized) and caloric consumption. The variable “working time as a farmer” was not included in the model due to multicollinearity with “age group”.

n = 359.

** n = 358.

*** n = 345.

**Table 8:** Factors associated with abdominal obesity among women in a rural Brazilian region.

Variables	Crude			Adjusted*		
	*P*-value	PR	95% CI	*P*-value	PR	95% CI
**Age group**						
Up to 29 years		1			1	
30 to 39 years	**0.015**	**1.81**	**(1.12–2.92)**	**0.007**	**1.73**	**(1.16–2.58)**
40 to 49 years	**< 0.001**	**2.49**	**(1.56–3.97)**	**< 0.001**	**2.19**	**(1.45–3.31)**
50 years or more	**< 0.001**	**2.55**	**(1.56–4.17)**	**< 0.001**	**2.16**	**(1.41–3.33)**
**Schooling**						
Less than 4 years		1			1	
4 to 8 years	**0.025**	**0.60**	**(0.39–0.94)**	0.533	0.89	(0.62–1.28)
More than 8 years	0.122	0.65	(0.37–1.12)	0.732	0.93	(0.60–1.43)
Transport used more frequently**						
Own vehicle		1			1	
On foot, by bicycle or bus	0.208	1.33	(0.85–2.09)	0.554	1.08	(0.84–1.39)
**Workload (hours/week)**						
Less than or equal to 40 hours		1			1	
More than 40 hours	0.058	0.74	(0.54–1.01)	**0.027**	**0.80**	**(0.65–0.97)**
**Number of places where they usually buy food**						
2 places or less		1			1	
3 places or more	0.155	1.25	(0.92–1.69)	**0.037**	**1.24**	**(1.01–1.51)**
**Monthly *per capita* expenditure on food purchases** ***						
R$ 100 or less		1			1	
> R$ 100 to < R$ 200	0.872	1.03	(0.74–1.44)	0.903	0.99	(0.79–1.23)
R$ 200 or more	0.148	1.37	(0.89–2.11)	0.374	1.12	(0.87–1.45)
**Pattern 1 –Local traditional**						
Largest adhesion (2^nd^ to 4^th^ quartiles)		1			1	
Least adhesion (1^st^ quartile)	**0.010**	**1.31**	**(1.07–1.61)**	**0.033**	**1.27**	**(1.02–1.57)**
**Pattern 2 –Traditional Brazilian**						
Largest adhesion (2^nd^ to 4^th^ quartiles)		1			1	
Least adhesion (1^st^ quartile)	**0.032**	**1.26**	**(1.02–1.55)**	**0.010**	**1.34**	**(1.07–1.67)**
**Pattern 3 –Industrialized**						
Least adhesion (1^st^ to 3^rd^ quartiles)		1			1	
Largest adhesion (4^th^ quartile)	0.108	0.75	(0.53–1.06)	0.378	0.85	(0.59–1.22)

PR, prevalence ratio; 95% CI, 95% confidence interval.

Poisson regression.

* Adjusted for all variables with *P* < 0.02 in the association test (age group, schooling, transport used more frequently, workload (hours/week), number of places where they usually buy food, monthly *per capita* expenditure on food purchases, dietary pattern 1 –Local traditional, dietary pattern 2 –Traditional Brazilian, and dietary pattern 3 –Industrialized) and caloric consumption. The variable “working time as a farmer” was not included in the model due to multicollinearity with “age group”.

n = 359.

** n = 358.

*** n = 345.

## Discussion

The present study identified a high prevalence of overweight status, obesity and abdominal obesity in this rural adult population, with higher rates in women. This scenario was also observed in other rural areas [24–26, 49, 50, 52–64], thus confirming that increased body weight in these regions is the main driver of the obesity epidemic in the world [19].

This finding is of concern due to the strong association of general and central obesity with the presence of NCDs [4, 5], especially cardiovascular diseases and diabetes [6, 8–11]. Such results break the paradigm that individuals who live in rural areas enjoy fully happy and healthy lifestyles, with healthy eating habits and adequate nutritional status, also called an “agrarian myth” [53]. Recent studies demonstrate that, in fact, apart from the problems related to the type of work activity performed, there is also a high prevalence of other chronic diseases in the field that are common to a more urbanized lifestyle, such as high blood pressure [28, 52, 54, 55, 65, 66], dyslipidemia [28, 54, 55], and diabetes mellitus [65–67]; all conditions closely related to general and abdominal obesity [6]. Specifically in this same population, we could already identify high prevalences of multimorbidity [27], cardiovascular risk [28], hypertension [29], depression [32, 33] and consumption to industrialized foods [30, 31], however, obesity had not yet been studied.

The prevalence rates of 31.5% overweight status, 19.7% obesity, 31.5% abdominal obesity and only 1.5% underweight status in these rural workers, despite belonging to a developing country, are consistent with the change in the nutritional status in rural areas of developed countries [56]. It is worth mentioning that the situation of underweight status, previously very common in rural areas, is declining in these regions [51, 56]. Obesity and overweight status in rural populations in Canada [24] and the United States of America [25, 63], as well as overweight status in rural workers in those countries [54, 61], corroborate the results of this article. However, the prevalence of overweight status in other developing countries, such as Sri Lanka [52], Zambia [68], Uganda [69], Tanzania [70] and China [71, 72], was lower than in the Brazilian population.

Few studies have evaluated the prevalence of abdominal obesity in rural populations worldwide, with reported rates of prevalence of 20.6% in India [73] to 59.2% in Sri Lanka [52]. In the region analyzed in this study, overweight status and abdominal obesity were slightly lower than the prevalence found in the South of Brazil [26, 58], similar to the Southeast [60] and higher than in the Midwest regions of the country [57]. Additionally, in women, the prevalence of obesity was higher than the national rural average (25.9% versus 21.8%) and very similar to abdominal obesity (49.9% versus 51.5%). The same occurred with men (13.9% versus 11.0% for obesity and 14.2% versus 14.8% for abdominal obesity) [49].

In this context, the most consistent finding with other investigations [50, 52, 57–60, 68, 73–76] is that obesity and abdominal obesity are more prevalent in women than in men. This demonstrates the health emergency, especially when we show in this rural population that the prevalence of general obesity is almost twice as much (25.9% versus 13.9%, *P* < 0.001) and abdominal obesity is more than triple (49.9% versus 14.2%, *P* < 0.001) in women than in men. Some authors seek to justify the higher prevalence of obesity in rural women. One of the main points discussed, mainly due to the greater prevalence of abdominal obesity in women, is their parity and biological conditions, such as hormonal changes and use of oral contraceptives [49, 74]. In addition, differences in occupations are mentioned, such as less demand for physical effort by women in rural activities, greater concentration in domestic work, and less leisure time [12, 19, 25, 49, 57, 77, 78]. These mentioned differences, as well as others identified in our work, can be even more notorious. While for men, factors related to income and the average time to purchase food remained associated with obesity and abdominal obesity, respectively, for women, other physiological factors (age) and lifestyle (adherence to dietary patterns, workload in the field, and places where food is purchased) were associated with body fat. Such findings may be related to cultural factors in some developing countries, in which the larger body can be understood as a sign of prosperity, good health and an excellent work tool [3], especially for men [72].

Apart from that, in men in this rural area, as mentioned, socioeconomic class was a factor related to a higher prevalence of obesity. In addition, a shorter average time to purchase food was associated with abdominal obesity. This second factor may be indirectly related to the individual’s income, since less time spent commuting to purchase food in remote areas, such as rural areas, correlates with the availability of vehicles, as well as a greater proximity to more urbanized centers in rural areas. In general, purchasing power linked to income seemed to be a factor related to obesity in rural regions, as evidenced in Canada [24], Uganda [69], India [79] and Bangladesh [74]. Similarly, in another rural area of Brazil, wealthier men were at higher risk for general obesity (PR = 1.7; 95% CI = 1.0–2.9) and central obesity (PR = 1.8; 95% CI = 1.1–2.9) [26].

For women, an important physiological factor, the older age group, was associated with a higher prevalence of obesity, both general and abdominal. This fact has also been described in the literature of other rural areas [26, 56, 59, 69, 72]. In Australian farmers, for example, it was found that the prevalence of overweight status and obesity peaked in the highest age groups, from 50–59 years old (66.4%) and 60–69 years old (69.6%) (*P* < 0.001) [56]. The physiological changes that occurred in the natural aging process, such as the loss of lean mass and hormonal and metabolic modifications, added to changes in lifestyle, and were capable of leading to an increase in excess weight in this population [26, 50, 75].

It is important to emphasize the role that body height can play in the risk of developing overweight and obesity, since it has already been described in the literature that short stature is associated with greater chances of obesity [80]. In other countries, the premature onset of puberty and, consequently, an earlier growth cessation process, can lead to the accumulation of fat mass in adulthood [81]. However, studies show a constant increase in the height of Brazilians, with the social environment and economic growth having a significant impact on this average height, in contrast to the variables race/color and place of residence (urban and rural), which did not prove to be determinants [82, 83]. In this sense, differences are still seen in the median height of adult Brazilians (on average 2.4 cm among men and 2.6 cm among women) in rural *versus* urban areas [15], however, the average of height in this rural population is more similar to the average height of Brazilian urban populations than others rural populations (1.75 cm for men and 1.61 cm for women).

Despite the importance of physiological factors, other lifestyle variables, both related to eating habits and indirect markers of physical activity related to work, can be related to the higher prevalence of obesity in females, as observed in our investigation. These factors may be linked to the “urbanization of the rural” [19], defined as the very accentuated presence of habits considered “urban” in rural environments, since, nowadays, agriculture and rural areas are increasingly mechanized, making it difficult to currently affirm that rural work is a protective factor for overweight persons in such populations [19, 84]. It is known that mechanized agricultural work increases the risk of obesity, mainly because it leads to lower energy expenditure rates than non-motorized tasks, demonstrating that, although the mechanization of agricultural work has obvious benefits in terms of productivity, its potential effects on the risks of overweight status and obesity should be recognized [61].

In this sense, in the present study, women who had a higher workload in the field had a lower prevalence of abdominal obesity, which was also found in a study of individuals from urban and rural areas of Bangladesh [78]. Although we have not directly measured mechanization in this rural area, the workload in the field can be an indicator of energy expenditure during work activities in this population [69, 78]. It is because the type of farming utilized (family farming, with intense participation of family members) and the very mountainous relief of this region prevent the use of large machinery in this countryside [85]. Moniruzzaman, Ahmed and Zaman [78] found that the energy expenditure for physical activity was almost double in the rural areas of an Asian country, with the main contributors to total physical activity found to be working hours and active commuting. In addition, Kirunda et al. [69] justified that the prevalence of overweight status in the rural areas of Uganda was lower due to the fact that rural residents are more actively involved in subsistence agriculture with intense labor and occupations that lead to less sedentary lifestyles, possibly inferring that non-mechanized rural activity is still a protective factor against being overweight.

Another factor identified in the present study, although rarely mentioned in the literature, especially with regard to women, is that lower adherence to traditional dietary patterns, both regional and national, was associated with a higher prevalence of both obesity and abdominal obesity. Globally, obesity is related to changes in eating habits, mainly due to the low consumption of fruit, vegetables and grains; continuous increase in consumption of processed foods; high intake of sugar drinks and other sugary products; and eating outside the home. Such changes, responsible for considerably increasing energy supply, clashes with human biology, creating a major change in body composition [12, 86].

As far as we know, this is the first population-based study that assessed adherence to dietary patterns determined *a posteriori* and its association with obesity and abdominal obesity in a rural area, taking into account the labor aspects of farmers. However, in the general population, some studies that evaluated dietary patterns using this method [87, 88], including meta-analyses [69, 70], also identified that greater adherence to healthier dietary patterns (often represented by traditional dietary patterns) was associated with a lower risk of obesity. Individuals in the highest categories of adherence to these healthy dietary patterns were 36% less likely to have general obesity (OR = 0.64; 95% CI = 0.52–0.78; *P* < 0.001) [90] and 19% less likely to have central obesity (OR = 0.81; 95% CI = 0.66–0.96; *P* < 0.001) [89]. Moreover, several meta-analyses identified that sex is a possible source of heterogeneity in the relationship between dietary patterns and obesity [89, 90]. This finding also justifies the permanence of the association of dietary patterns with obesity in women, but not in men from the Brazilian region evaluated in this study. Finally, also in agreement with our findings, unhealthy dietary patterns had no significant association with abdominal obesity [88, 89].

It is important to mention that other studies that evaluated food consumption in rural areas identified that many individuals in these regions maintained their consumption of traditional foods, such as rice, breads, leafy beans, beans, cow’s milk, animal fats, margarine, sugar, cassava flour and coffee [30, 57, 91–93]. However, it is also possible to notice an increase in consumption of industrialized foods [30, 31, 93–96], which demonstrates that factors associated with globalization also affect eating habits in rural areas [30, 66, 94–97]. Although agricultural residents have options to obtain their food from their plantations or through community sharing, they may have limited availability of healthy food due to the long distances from places that sell food, in addition to the high prices of some items in small local markets, low availability of transport for access to cheaper products and difficulty of storing these foods [98, 99]. Specifically for rural workers, it is possible that the food produced is understood to be goods for sale to earn income, not perceived as products for self-consumption [100].

This change in habits, most likely due to the consumption of industrialized foods, occurs mainly in younger individuals [30, 94]. Popkin, Adair and Ng [12] argue that dietary changes may not occur equally in all age groups, as individuals respond differently to social and economic modifications, and it tends to be the younger generation that adopts new dietary patterns more quickly, while older individuals continue to eat in more traditional–and sometimes healthier–ways. Older individuals are more motivated to change their eating habits due to the emergence of diseases and, thus, select more nutritious and healthy products in their diet [101]. Due to these processes, which have not been detectable in cross-sectional studies, there is justification for the non-association of greater adherence to the “industrialized” dietary pattern with obesity in this rural region, since younger individuals are the ones who mostly adhere to the consumption of these foods without manifesting the consequences of their eating habits, such as obesity. Although there is a clear increase in excess weight with increasing age in most diverse populations, this growth is also very prevalent in young adults, who are considered a “vulnerable group” to obesity due to their unhealthy lifestyles [3]. It is important to mention a study carried out between 2009 and 2010 with children aged 7 to 10 years in the same city as the present study. At that time, it was already possible to identify a 5% prevalence of obesity [102]. A more recent study in another Brazilian region identified that indigenous children are shorter in stature compared to urban and rural children, with children having high rates of excess weight in all regions, demonstrating that poor eating habits and a sedentary lifestyle do not were characteristic only of urban centers [103]. This fact is reinforced by national data, in which there is a prevalence of obesity of 10.1% among Brazilian children aged until 5 years, 14.7% among adolescent girls and 15.4% among adolescent boys, emphasizing that obesity can develop in childhood, and this perspective of family nutritional status is a valuable starting point for public health programs [104].

We highlight that the cross-sectional design may be a limitation of this work since factors related to the time of exposure may not have been identified as factors related to a higher prevalence of obesity in the analyzed group. Moreover, BMI, used to assess the prevalence of general obesity, may have limitations regarding the assessment of nutritional status on an individual level, especially when comparing individuals of different age groups or levels of physical activity. However, its concomitant evaluation with abdominal obesity aims to reduce this possible bias, since WC is closely related to visceral fat content and, therefore, to metabolic risks [8]. Furthermore, BMI and WC are biological markers and risk factors for cardiovascular diseases that have mostly been adopted in majority epidemiological studies, in addition to being used by the WHO, precisely because they are simple and low cost measures [6, 8] that allow for the standardization of data comparison in different countries, as well as over time [1, 54]. When evaluated together, they can predict the risk of several chronic diseases [4–11].

We conclude that the prevalence of overweight status, obesity and abdominal obesity are high in this rural area of Brazil, with higher rates in women. In men, a higher prevalence of obesity and abdominal obesity was associated with a greater socioeconomic class and shorter commuting time to purchase food. In women, the factors related to a higher prevalence of obesity were age, lower adherence to traditional “local” and “Brazilian” dietary patterns, as well as the decreased workload in the field and the higher number of places to buy food.

Such findings demonstrate that the “agrarian myth” must be deconstructed so that obesity is also considered an issue in the health care of remote and rural populations. Given the available data, there is a need to increase the practice of physical activity in rural areas, especially related to leisure activities, in addition to the need to improve eating habits and promoting healthier eating environments for individuals, while respecting the traditional food culture, especially to contain the advancement of abdominal obesity in women. Finally, due to the multicausal nature of obesity, coping strategies for this condition must include a multiple, intersectoral and interdisciplinary approach. It is also necessary to take into account that rural areas are not homogeneous and require personalized situations to practice wholesome habits in order to control the increase in obesity and abdominal obesity in these populations, especially in women, the most vulnerable group.

## Supporting information

S1 Dataset (SAV)
